# Chemotherapy: United Kingdom National Multidisciplinary Guidelines

**DOI:** 10.1017/S0022215116000840

**Published:** 2016-05

**Authors:** C G Kelly

**Affiliations:** Northern Centre for Cancer Care and Newcastle University, Freeman Hospital, Newcastle upon Tyne, UK

## Abstract

This is the official guideline endorsed by the specialty associations involved in the care of head and neck cancer patients in the UK. This paper summarises the role of chemotherapy in head and neck cancer management, recent advances and what the future holds for this modality.

## Introduction

Chemotherapy alone cannot cure head and neck cancer. It is used in conjunction with other treatments, surgery and radiotherapy (RT), to improve outcomes in terms of local control, organ preservation with continued organ function and to decrease the incidence of subclinical micro-metastatic spread.

Chemotherapy is not given routinely for early primary T1/T2 disease without nodal involvement. Chemotherapy is given for its direct tumouricidal effect, at both the local primary and distant metastatic sites. If given with RT it can have a radio sensitising effect, making cancer cells more susceptible to RT and increasing the cancer cell kill. It may be used as induction chemotherapy (ICT), almost always before RT rather than surgery. If ICT is used, further chemotherapy is usually given with subsequent RT, and this is known as sequential chemotherapy. More commonly, chemotherapy is given only concurrently with radiotherapy (combined chemoradiotherapy) with only a minority of patients having induction or sequential regimens. Combined chemoradiotherapy has been shown to improve local control and increase survival where primary surgery has been the definitive treatment in selected populations.

## Induction (neoadjuvant) chemotherapy

The response to neoadjuvant chemotherapy could give important prognostic information, as it can act as a surrogate marker for response to later treatment.

This latter advantage was used in one of the earliest trials of neoadjuvant chemotherapy for organ preservation, the ‘Veterans’ trial,^1^ where patients were given two cycles of cisplatin and 5-fluorouracil (5-FU), and if there was a response to chemotherapy patients went on to have chemotherapy and RT, but if there was no response, the patients went directly to laryngectomy.

Induction chemotherapy is considered beneficial for several reasons. If ICT could shrink primary tumour volumes before the principal treatment of RT or chemoradiotherapy, this might allow better blood flow into the tumour allowing a greater tumouricidal dose of drugs into the tumour and decrease the volume of hypoxic areas which would decrease the radio-resistance that hypoxic cancer cells show. Improved local control would lead to a greater chance of organ preservation and functionality. Since surgery and RT are both locoregional treatments, ICT could theoretically treat distant subclinical metastatic disease. The response to ICT could give important prognostic information, as it can act as a surrogate marker for response to later treatment.

One of the main evidence sources for the use of chemotherapy in head and neck cancer is the Meta-analysis of Chemotherapy in Head and Neck Cancer (MACH-NC) which was originally published in 2000 and updated in 2007 and 2009.^2^ This overview reviewed 87 trials containing data on over 16 000 patients, with overall survival as the primary endpoint. There was no overall survival benefit with the use of ICT when compared with primary surgery or RT alone, although cisplatin and 5-FU delivered as combined chemoradiotherapy did show some benefit. It also suggested that ICT may reduce the incidence of distant metastases more effectively than combined chemoradiotherapy.

Debate continues as to whether ICT followed by combined chemoradiotherapy is more beneficial than combined chemoradiotherapy alone. Some large trials, such as the Spanish Head and Cancer Corporative Group trial have shown no benefit,^3^ while others, such as a large Italian trial comparing ICT followed by combined chemoradiotherapy to combined chemoradiotherapy alone showed significantly improved overall survival for the former arm.^4^ Interest was rekindled in ICT when two trials, one European, and one from North America, TAX 323^5^ and TAX 324^6^ showed a benefit by including a taxane, such as docetaxel or paclitaxel in the chemotherapy regimen in addition to cisplatin and 5-FU. The evidence suggested that adding a taxane, such as docetaxel or paclitaxel to cisplatin and 5-FU, i.e. docetaxel/cisplatin/5-FU (TPF) *vs* cisplatin/5-FU (PF) did improve survival in the TPF arm, but at a cost of much higher toxicity. However, these trials have been criticised for not using optimal concurrent chemotherapy schedules. Based on further phase 3 studies (DeCIDE,^7^ PARADIGM trial and^8^ TREMPLIN study^9^) the evidence to date does not suggest ICT is in general beneficial in head and neck cancer.

Usually, induction and sequential regimens are offered to patients with good performance status, fewer comorbidities and those with bulky nodal disease, stage N2b and above, and where surgery is not appropriate.

## Concurrent or concomitant chemotherapy

The main advantage of combined chemoradiotherapy, over sequential chemotherapy, is the reduced chance of patients having to stop treatment because of toxicity, and resulting in breaks in RT, which is radiobiologically suboptimal and can be detrimental to treatment outcome.

In the MACH-NC trial,^2^ the use of combined chemoradiotherapy showed that it gave a survival benefit when added to RT alone, giving a 6.5 per cent decrease in mortality at five years, in absolute terms. This benefit was not seen in patients over 70 years of age. The most commonly used combined chemoradiotherapy regimens are cisplatin 100 mg/m^2^ on days 1, 22 and 43 of RT, either alone or with 5-FU, 1 g/day on days 1–4, and then repeated 3 weekly with cisplatin. If 5-FU is added, the cisplatin dose is usually reduced. Although this regimen is commonly used, there are few direct comparisons with other combined chemoradiotherapy within randomised controlled trials.

Increased toxicity produced by adding platinum chemotherapy to RT can be considerable. A significant proportion of patients do not receive all three cycles of chemotherapy because of toxicity, but one study has shown no survival difference in patients receiving two cycles of cisplatin rather than three cycles, but the RT given was not identical within the arms of this study.^10^ chemotherapy toxicity can also interfere detrimentally with RT delivery causing breaks during treatment which are associated with poorer outcomes.

If cisplatin is contraindicated because of renal function status, the presence of neuropathy, tinnitus or deafness, or where there is a danger of fluid overload with the necessary pre-hydration used in cisplatin administration, carboplatin can be considered as it causes less nephrotoxicity, ototoxicity and peripheral neuropathy but is more myelosuppressive. It is not thought to be as tumouricidal as cisplatin and for this reason it has now been largely overtaken by the epidermal growth factor receptor inhibitor, cetuximab in clinical practice when cisplatin is contraindicated.

## Concurrent radio-sensitisers

It is known that tumour cell hypoxia induces radioresistance, and there has been renewed interest in giving hypoxic cell radiosensitising drugs during RT. The two most common in use are the antihelminthic drug nimorazol, which is extensively used in some parts of Europe, and tirapazamine, an anticoagulant which is activated in hypoxic environments. Although established in some parts of the world, trials are ongoing with these agents to establish efficacy and with nimorazol, patient tolerability.

## Chemotherapy and human papilloma virus (HPV)-positive tumours

Human papilloma virus is known to have an aetiological role in inducing some head and neck cancers, especially in the oropharynx where HPV infection may be linked to 50–80 per cent of tumours. There is evidence from several studies that outcomes are better following treatment in patients with HPV-positive tumours. There is also growing evidence that continuing to smoke negates the outcome benefit associated with HPV positivity.

Given the good prognosis, the question arises if HPV-positive cancers are being overtreated with standard head and neck chemoradiotherapy regimens and being given unnecessary morbidity. At present there is not enough evidence to alter chemotherapy or indeed RT treatment regimens depending on the patient's HPV status, outside of the context of a clinical trial.

Several trials are now investigating these questions, most using cetuximab comparing with cisplatin (see below). These include the RTOG 1016 in the USA, the De-ESCALATE HPV study in the UK and the Trans-Tasman Radiotherapy Group 12.01 study in Australia.

## Targeted biological agents

Targeted therapy in head and neck cancer developed with the recognition that epidermal growth factor receptor (EGFR) is overexpressed in the majority of head and neck cancers, up to 90 per cent in some studies, and is associated with a poorer prognosis. When a growth factor attaches to its receptor on the cell surface, cells are stimulated to divide and consequently tumours grow. If the receptor is abnormal because of a mutation the stimulation to divide may even occur without growth factors interacting with the receptor. Cetuximab is a mouse–human chimaeric monoclonal antibody which binds to the extracellular portion of EGFR and turns this signalling system off.

In the initial innovative cetuximab trial by Bonner *et al*.,^11^ patients with advanced head and neck cancer were randomised to receive radical RT with or without cetuximab. At three years, survival (55 *vs* 45 per cent) and local control (50 *vs* 41 per cent) was better in the patient group who had received cetuximab.^12^ Although these initial results were encouraging, a major drawback to the study was that, since the study had started, RT alone as used in this study had been overtaken as a standard of care by combined chemoradiotherapy. So, comparing radiation alone *vs* radiation plus cetuximab was much less relevant in the context of contemporary standard practice. Also in the initial trial patients were not stratified by HPV status.

Despite initial hopes that cetuximab would give less toxicity than the standard chemotherapy, and can therefore be given to older patients and those with a poorer performance status, it has been shown to have a different, although not necessarily less toxic, morbidity profile in the form of grade 3 and 4 radiation dermatitis. Patients may also develop an acne-like rash predominantly over the face, neck and trunk with a more eczema-like condition at the fingertips and elbows. In a minority of patients this reaction can be so severe that cetuximab may need to be stopped as these side effects can usually be managed by increasing the treatment interval and supportive care with topical medications. There is some suggestion that patients who develop this rash may also have a better tumour response with improved overall survival.

Other targeted EGFR monoclonal antibodies are under investigation such as panitumamab or zalutumumab, but to date with less encouraging results showing no improvement in overall survival.

Another potential target further down this biological pathway, offering a different mechanism of action is used by erlotinib, a small molecule inhibitor of EGFR tyrosine kinase. One phase II trial of erlotinib given alone or combined with cisplatin, unfortunately did not show any benefit in outcome for the combination. Despite this other targets in the epidermal growth factor receptor pathway are being investigated.

## Chemotherapy for recurrent or metastatic head and neck cancer

Chemotherapy or targeted biological agents may be indicated for patients with recurrent and/or metastatic disease but prognosis for patients with metastatic disease has a median survival of approximately 6–12 months in most studies.

Appropriateness of chemotherapy depends on several factors such as extent and burden of disease; whether symptoms are present or not; whether failure of control has taken place at the primary site only; and whether there is metastatic disease only or both. The most important factor often is the fitness and performance status of the patient and whether they could tolerate the proposed chemotherapy, and how much it would reduce their pre-treatment quality of life, for whatever limited survival period they have.

## Locoregional failure

In this group of patients where salvage surgery or retreatment with RT or combined chemoradiotherapy is being considered, it is important to be aware if distant metastatic disease is also present and also to establish that the locoregional recurrent disease is not a second primary head and neck cancer. Discovering that metastatic disease is also present is not an absolute contraindication to salvage treatments at the primary site, as locoregional failure and metastatic disease can be considered as two separate problems in the patient's management plan. If good palliation at the primary site or locoregionally can be achieved relatively easily, by a salvage procedure, the presence of metastatic disease, especially small volume metastatic disease, should not necessarily stop treatment to the primary or locoregional site. The patients who do better with salvage treatment are those with smaller volumes of recurrence, a longer disease-free interval and less comorbidity. Some particular head and neck subsites such as the larynx, also have better outcomes.^13^

## Distant metastases

Chemotherapy is often indicated here, as part of a best supportive care package to improve symptoms, but has not been shown to significantly extend survival. The therapeutic window for giving chemotherapy in this situation would be when the patient still has an appropriate performance status to receive and benefit from chemotherapy, with the trade-off being, an improved symptom state for the inevitable morbidity caused by the chemotherapy. The choice of regimen depends on factors such as performance status, comorbidities, renal function, estimated physiological reserve of the patient and the interval since last chemotherapy.

If chemotherapy is to be given for distant metastatic disease then which regimen is most appropriate depends on several factors including performance status, comorbidities present, renal function and the estimated physiological reserve of the patient. Also which regimens the patients had before and the interval since last chemotherapy may be important.

The most common regimens used are cisplatin or carboplatin with 5-FU. These give an expected response rate of approximately 30 per cent. Carboplatin is used more in this palliative metastatic setting than with induction or concurrent regimens, because although deemed slightly less effective than cisplatin; its less toxic side-effect profile, can be seen to be more appropriate in the palliative setting. Elderly patients do appear to respond to platinum-based chemotherapy in the metastatic setting,^14^ in contrast to a lack of benefit in the elderly when used in primary chemoradiotherapy regimens. Other more toxic chemotherapy regimens have also been investigated using platinum and a taxane (docetaxel or paclitaxel), in combination, but no survival benefit has been demonstrated.

Cetuximab added to cisplatin and 5-FU, can increase both response rate and improve short-term survival slightly as shown in the EXTREME trial,^15^ but five-year follow-up published recently in abstract form shows very low survival for patients in both arms of the study. The EXTREME study did not allow crossover between regimens, so similar results might be achieved by the use of cisplatin and 5-FU followed by cetuximab used sequentially. In patients who have become refractive to cisplatin and 5-FU, cetuximab as a single agent does have a low response rate of approximately 10–15 per cent.^16^^,^^17^

### Key points

• Concurrent chemoradiotherapy is at present the standard of care for treatment of locally advanced head and neck cancer with a confirmed survival benefit of 6.5 per cent at five years
• Single agent cisplatin, which in the past has been shown to be as effective as multiple drug regimes, is now being challenged by the introduction of the use of taxanes
• Targeted biological agents, such as cetuximab, have a role to play in both advanced head and neck cancer and recurrent or metastatic disease but those roles are still being established
• At present human papilloma virus status does not alter management regimens, although there are multiple studies underway examining if less intense treatment, both with radiotherapy and chemotherapy, could be given to achieve the same outcome but with less toxicity
• The potential benefit of neoadjuvant or induction chemotherapy is being re-examined now, but most recent work has not shown a substantial benefit
• Elderly patients benefit least in terms of survival advantage with the use of concurrent chemotherapy.
